# Tetra­imidazolium hexa-μ_4_-oxido-dodeca-μ_2_-oxido-dodeca­oxidohexa­arsenate(III)hexa­molybdenum(VI)cuprate(II)

**DOI:** 10.1107/S1600536810037414

**Published:** 2010-09-30

**Authors:** Meiduo Liu, Zhanhua Su, Yongchen Shang

**Affiliations:** a College of Chemistry and Chemical Engineering, Harbin Normal University, Harbin, People’s Republic of China; b Department of Safety and Environmental Engineering, Jixi University, Jixi, People’s Republic of China

## Abstract

The title compound, (C_3_H_5_N_2_)_4_[As_6_CuMo_6_O_30_], is made up of a centrosymmetric anionic cluster and four imidazolium cations. In the cluster, the central Cu^II^ atom is six-coordinated and lies on an inversion center. Adjacent clusters are linked *via* N—H⋯O hydrogen bonds between the imidazole cations and polyoxidoanions into a three-dimensional supra­molecular architecture.

## Related literature

For general background to polyoxidometalates, see: Müller *et al.* (1998 ▶). For general background to molybdoarsenates, see: Fidalgo *et al.* (2002 ▶); Sun *et al.* (2007 ▶).
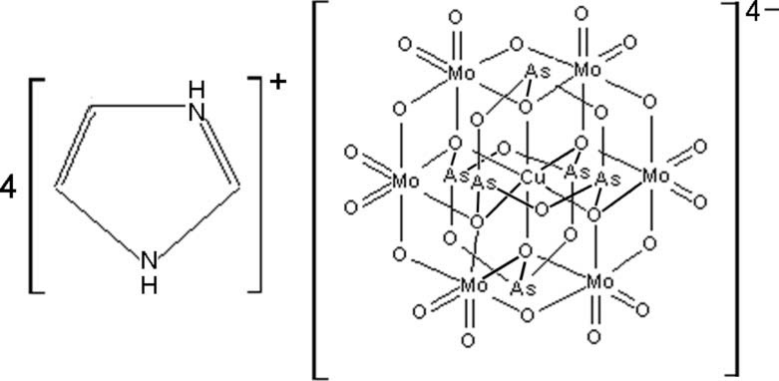


## Experimental

### 

#### Crystal data

(C_3_H_5_N_2_)_4_[As_6_CuMo_6_O_30_]
*M* *_r_* = 1845.07
Monoclinic, 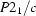

*a* = 10.5696 (7) Å
*b* = 19.2842 (12) Å
*c* = 10.4678 (7) Å
β = 106.747 (1)°
*V* = 2043.1 (2) Å^3^
*Z* = 2
Mo *K*α radiation
μ = 7.22 mm^−1^
*T* = 298 K
0.28 × 0.25 × 0.20 mm

#### Data collection

Bruker APEXII CCD diffractometer
Absorption correction: multi-scan (*SADABS*; Bruker, 2001 ▶) *T*_min_ = 0.237, *T*_max_ = 0.326
12666 measured reflections
4906 independent reflections
4057 reflections with *I* > 2σ(*I*)
*R*_int_ = 0.029

#### Refinement

*R*[*F* ^2^ > 2σ(*F* ^2^)] = 0.030
*wR*(*F* ^2^) = 0.073
*S* = 1.03
4906 reflections
286 parameters
1 restraint
H-atom parameters constrained
Δρ_max_ = 0.56 e Å^−3^
Δρ_min_ = −1.94 e Å^−3^

### 

Data collection: *APEX2* (Bruker, 2007 ▶); cell refinement: *SAINT* (Bruker, 2007 ▶); data reduction: *SAINT*; program(s) used to solve structure: *SHELXS97* (Sheldrick, 2008 ▶); program(s) used to refine structure: *SHELXL97* (Sheldrick, 2008 ▶); molecular graphics: *SHELXTL* (Sheldrick, 2008 ▶); software used to prepare material for publication: *SHELXTL*.

## Supplementary Material

Crystal structure: contains datablocks I, global. DOI: 10.1107/S1600536810037414/hy2352sup1.cif

Structure factors: contains datablocks I. DOI: 10.1107/S1600536810037414/hy2352Isup2.hkl

Additional supplementary materials:  crystallographic information; 3D view; checkCIF report

## Figures and Tables

**Table 1:** Hydrogen-bond geometry (Å, °)

*D*—H⋯*A*	*D*—H	H⋯*A*	*D*⋯*A*	*D*—H⋯*A*
N1—H1*A*⋯O4^i^	0.86	1.81	2.664 (5)	173
N2—H2*A*⋯O3	0.86	1.99	2.748 (5)	146
N2—H2*A*⋯O9^ii^	0.86	2.42	3.020 (5)	127
N3—H3*A*⋯O7^ii^	0.86	2.09	2.867 (6)	150
N4—H4*A*⋯O2^iii^	0.86	2.00	2.834 (6)	165

Symmetry codes: (i) 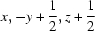; (ii) 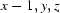; (iii) 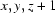.

**Fig. 1.**
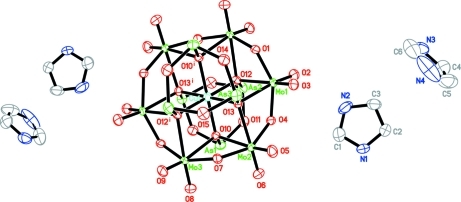
The molecular structure of the title compound. Displacement ellipsoids are drawn at the 50% probability level. H atoms have been omitted. [Symmetry code: (i) 2-x, -y, -z.]

## supplementary crystallographic information

### Comment

Polyoxometalates have unusual structural chemistry and properties that make them attractive for applications in materials science, electrochemical, catalysis and photochemical (Müller *et al.*, 1998). Molybdenum arsenates are an important part in this field. So far the reports on molybdenum arsenates have been mainly concentrated on several discrete molybdenum arsenate clusters (Fidalgo *et al.*, 2002; Sun *et al.*, 2007). Therefore, further research is necessary to enrich and develop this branch. We try to obtain new materials based on inorganic molybdenum arsenate with novel structures. Here, the synthesis and crystal structure of the title compound is reported.

The structure of the title compound is shown in Fig. 1. The asymmetric unit consists of two protonated imidazole cations and a half cluster anion. The anion is centrosymmetric with the Cu^II^ atom lying on an inversion center. The cluster is derived from the A-type Anderson anion, in which a central CuO_6_ octahedron is coordinated with six MoO_6_ octahedra hexagonally arranged by sharing their edges in a plane. Two cyclic As_3_O_3_ trimers are capped on the opposite faces of the Anderson-type anion plane. The four free protonated imidazole molecules act as charge compensating cations. The adjacent clusters are linked *via* N—H···O hydrogen bonds between the imidazole cations and polyoxoanions into a three-dimensional supramolecular architecture (Table 1).

### Experimental

A mixture of hexaammonium heptamolybdate tetrahydrate (1.11 g, 0.89 mmol), sodium arsenite (0.41 g, 3.03 mmol), cupric chloride (0.20 g, 1.17 mmol), imidazole (0.27 g, 4.01 mmol) and water (20 ml) was placed in a 30 ml Teflon-lined Parr bomb. The bomb was heated to 413 K for 5 d. Blue block shaped crystals were isolated from the cooled solution in a 72% yield based on Mo. Analysis, calculated for C_12_H_20_As_6_CuMo_6_N_8_O_30_: C 7.81, H 1.09, N 6.07%; found: C 7.83, H 1.13, N 6.04%.

### Refinement

H atoms were positioned geometrically and refined using a riding model, with C—H = 0.93 and N—H = 0.86 Å, and with *U*_iso_(H) = 1.2*U*_eq_(C, N).

### Crystal data

**Table:**

(C_3_H_5_N_2_)_4_[As_6_CuMo_6_O_30_]	*F*(000) = 1734
*M**_r_* = 1845.07	*D*_x_ = 2.999 Mg m^−^^3^
Monoclinic, *P*2_1_/*c*	Mo *K*α radiation, λ = 0.71073 Å
Hall symbol: -P 2ybc	Cell parameters from 4555 reflections
*a* = 10.5696 (7) Å	θ = 2.3–28.1°
*b* = 19.2842 (12) Å	µ = 7.22 mm^−^^1^
*c* = 10.4678 (7) Å	*T* = 298 K
β = 106.747 (1)°	Block, blue
*V* = 2043.1 (2) Å^3^	0.28 × 0.25 × 0.20 mm
*Z* = 2	

### Data collection

**Table:**

Bruker APEXII CCD diffractometer	4906 independent reflections
Radiation source: fine-focus sealed tube	4057 reflections with *I* > 2σ(*I*)
graphite	*R*_int_ = 0.029
φ and ω scans	θ_max_ = 28.3°, θ_min_ = 2.3°
Absorption correction: multi-scan (*SADABS*; Bruker, 2001)	*h* = −13→14
*T*_min_ = 0.237, *T*_max_ = 0.326	*k* = −25→19
12666 measured reflections	*l* = −13→13

### Refinement

**Table:**

Refinement on *F*^2^	Primary atom site location: structure-invariant direct methods
Least-squares matrix: full	Secondary atom site location: difference Fourier map
*R*[*F*^2^ > 2σ(*F*^2^)] = 0.030	Hydrogen site location: inferred from neighbouring sites
*wR*(*F*^2^) = 0.073	H-atom parameters constrained
*S* = 1.03	*w* = 1/[σ^2^(*F*_o_^2^) + (0.0338*P*)^2^ + 2.1525*P*] where *P* = (*F*_o_^2^ + 2*F*_c_^2^)/3
4906 reflections	(Δ/σ)_max_ = 0.001
286 parameters	Δρ_max_ = 0.56 e Å^−^^3^
1 restraint	Δρ_min_ = −1.94 e Å^−^^3^

### Fractional atomic coordinates and isotropic or equivalent isotropic displacement parameters (Å^2^)

**Table:**

	*x*	*y*	*z*	*U*_iso_*/*U*_eq_	
Mo1	0.73132 (3)	0.054936 (18)	0.07751 (3)	0.01939 (9)	
Mo2	1.01930 (3)	0.143475 (18)	0.18894 (3)	0.02087 (9)	
Mo3	1.29116 (3)	0.084229 (18)	0.11868 (4)	0.02065 (9)	
Cu1	1.0000	0.0000	0.0000	0.02300 (16)	
As1	1.03800 (5)	0.16004 (2)	−0.13845 (4)	0.02655 (11)	
As2	0.75566 (4)	0.08081 (2)	−0.24782 (4)	0.02711 (11)	
As3	0.98689 (4)	−0.02215 (2)	0.32318 (4)	0.02600 (11)	
O1	0.7044 (3)	−0.04164 (14)	0.0461 (3)	0.0222 (6)	
O2	0.5905 (3)	0.09037 (16)	−0.0244 (3)	0.0324 (7)	
O3	0.7135 (3)	0.05775 (16)	0.2348 (3)	0.0301 (7)	
O4	0.8381 (3)	0.13750 (14)	0.0796 (3)	0.0222 (6)	
O5	0.9982 (3)	0.14957 (17)	0.3446 (3)	0.0335 (7)	
O6	1.0471 (3)	0.22633 (16)	0.1453 (3)	0.0347 (7)	
O7	1.1939 (3)	0.10410 (15)	0.2460 (3)	0.0240 (6)	
O8	1.3348 (3)	0.16566 (16)	0.0847 (3)	0.0355 (8)	
O9	1.4277 (3)	0.05057 (17)	0.2328 (3)	0.0330 (7)	
O10	1.0708 (3)	0.10168 (14)	0.0027 (3)	0.0194 (6)	
O11	0.8615 (3)	0.15371 (15)	−0.1931 (3)	0.0311 (7)	
O12	0.8071 (3)	0.02681 (14)	−0.1035 (3)	0.0190 (6)	
O13	0.9515 (3)	0.02733 (14)	0.1712 (3)	0.0193 (6)	
O14	0.9234 (3)	−0.10364 (16)	0.2570 (3)	0.0294 (7)	
O15	1.1601 (3)	−0.03719 (17)	0.3501 (3)	0.0306 (7)	
C1	0.7300 (6)	0.1970 (3)	0.4181 (6)	0.0547 (16)	
H1	0.8100	0.1911	0.3989	0.066*	
C2	0.5755 (5)	0.2391 (3)	0.4939 (5)	0.0402 (12)	
H2	0.5301	0.2679	0.5369	0.048*	
C3	0.5305 (6)	0.1807 (3)	0.4292 (6)	0.0481 (14)	
H3	0.4477	0.1610	0.4181	0.058*	
C4	0.2581 (6)	0.1681 (3)	0.5787 (6)	0.0498 (14)	
H4	0.2056	0.2032	0.5297	0.060*	
C5	0.3261 (7)	0.1725 (4)	0.7012 (7)	0.072 (2)	
H5	0.3326	0.2109	0.7565	0.087*	
C6	0.3535 (7)	0.0703 (4)	0.6296 (9)	0.072 (2)	
H6	0.3815	0.0249	0.6254	0.086*	
N1	0.6994 (4)	0.2487 (2)	0.4856 (5)	0.0422 (11)	
H1A	0.7501	0.2831	0.5190	0.051*	
N2	0.6282 (5)	0.1554 (2)	0.3826 (5)	0.0511 (13)	
H2A	0.6240	0.1179	0.3369	0.061*	
N3	0.2742 (4)	0.1053 (3)	0.5320 (5)	0.0493 (12)	
H3A	0.2388	0.0906	0.4521	0.059*	
N4	0.3864 (5)	0.1108 (5)	0.7346 (6)	0.091 (3)	
H4A	0.4378	0.0998	0.8118	0.109*	

### Atomic displacement parameters (Å^2^)

**Table:**

	*U*^11^	*U*^22^	*U*^33^	*U*^12^	*U*^13^	*U*^23^
Mo1	0.01533 (17)	0.02100 (17)	0.02186 (18)	0.00083 (13)	0.00538 (13)	−0.00261 (13)
Mo2	0.01802 (18)	0.02131 (18)	0.02259 (18)	−0.00029 (14)	0.00474 (14)	−0.00575 (13)
Mo3	0.01496 (17)	0.02128 (18)	0.02509 (19)	−0.00293 (13)	0.00477 (14)	−0.00520 (13)
Cu1	0.0210 (4)	0.0247 (4)	0.0227 (4)	−0.0005 (3)	0.0052 (3)	−0.0014 (3)
As1	0.0304 (3)	0.0220 (2)	0.0268 (2)	−0.00166 (18)	0.00755 (19)	0.00428 (17)
As2	0.0213 (2)	0.0327 (2)	0.0245 (2)	0.00458 (18)	0.00205 (18)	0.00643 (18)
As3	0.0291 (2)	0.0318 (2)	0.0175 (2)	0.00211 (19)	0.00728 (18)	0.00140 (17)
O1	0.0210 (15)	0.0219 (14)	0.0254 (15)	−0.0049 (12)	0.0095 (12)	−0.0016 (11)
O2	0.0228 (16)	0.0330 (18)	0.0384 (18)	0.0045 (13)	0.0038 (14)	−0.0019 (14)
O3	0.0300 (17)	0.0327 (17)	0.0309 (17)	−0.0025 (14)	0.0142 (14)	−0.0056 (13)
O4	0.0188 (14)	0.0185 (14)	0.0270 (15)	0.0012 (11)	0.0029 (12)	−0.0012 (11)
O5	0.0263 (17)	0.046 (2)	0.0284 (17)	0.0003 (14)	0.0087 (14)	−0.0085 (14)
O6	0.0355 (18)	0.0233 (16)	0.045 (2)	−0.0010 (14)	0.0103 (15)	−0.0075 (14)
O7	0.0185 (14)	0.0308 (16)	0.0209 (14)	−0.0014 (12)	0.0028 (11)	−0.0075 (12)
O8	0.0338 (18)	0.0262 (16)	0.050 (2)	−0.0092 (14)	0.0182 (16)	−0.0082 (15)
O9	0.0175 (15)	0.0403 (19)	0.0362 (18)	0.0017 (14)	−0.0005 (13)	−0.0076 (14)
O10	0.0177 (14)	0.0203 (14)	0.0196 (14)	−0.0009 (11)	0.0046 (11)	−0.0005 (11)
O11	0.0302 (17)	0.0274 (16)	0.0338 (18)	0.0048 (13)	0.0063 (14)	0.0065 (13)
O12	0.0150 (13)	0.0215 (14)	0.0190 (14)	−0.0002 (11)	0.0028 (11)	0.0025 (11)
O13	0.0185 (14)	0.0213 (14)	0.0178 (13)	0.0018 (11)	0.0049 (11)	0.0000 (11)
O14	0.0324 (17)	0.0286 (16)	0.0296 (17)	−0.0015 (13)	0.0126 (14)	0.0037 (13)
O15	0.0294 (17)	0.0382 (18)	0.0213 (15)	0.0017 (14)	0.0028 (13)	0.0038 (13)
C1	0.052 (4)	0.047 (3)	0.076 (4)	−0.002 (3)	0.036 (3)	−0.008 (3)
C2	0.038 (3)	0.036 (3)	0.050 (3)	0.000 (2)	0.017 (2)	−0.010 (2)
C3	0.048 (3)	0.048 (3)	0.053 (3)	−0.021 (3)	0.021 (3)	−0.017 (3)
C4	0.052 (4)	0.044 (3)	0.048 (3)	0.011 (3)	0.005 (3)	0.010 (3)
C5	0.073 (5)	0.084 (5)	0.059 (4)	−0.036 (4)	0.018 (4)	−0.023 (4)
C6	0.062 (5)	0.054 (4)	0.112 (7)	0.019 (3)	0.045 (5)	0.049 (4)
N1	0.037 (2)	0.028 (2)	0.061 (3)	−0.0103 (18)	0.014 (2)	−0.0152 (19)
N2	0.074 (4)	0.032 (2)	0.057 (3)	−0.012 (2)	0.034 (3)	−0.019 (2)
N3	0.038 (3)	0.055 (3)	0.048 (3)	−0.009 (2)	0.000 (2)	−0.004 (2)
N4	0.034 (3)	0.190 (8)	0.041 (3)	0.001 (4)	0.000 (3)	0.060 (4)

### Geometric parameters (Å, °)

**Table:**

Mo1—O2	1.704 (3)	As2—O11	1.785 (3)
Mo1—O3	1.711 (3)	As2—O12	1.786 (3)
Mo1—O1	1.898 (3)	As2—O15^i^	1.788 (3)
Mo1—O4	1.948 (3)	As3—O14	1.770 (3)
Mo1—O13	2.312 (3)	As3—O15	1.793 (3)
Mo1—O12	2.324 (3)	As3—O13	1.800 (3)
Mo2—O6	1.710 (3)	C1—N2	1.308 (7)
Mo2—O5	1.711 (3)	C1—N1	1.316 (7)
Mo2—O7	1.925 (3)	C1—H1	0.9300
Mo2—O4	1.931 (3)	C2—C3	1.329 (7)
Mo2—O10	2.315 (3)	C2—N1	1.351 (6)
Mo2—O13	2.343 (3)	C2—H2	0.9300
Mo3—O8	1.703 (3)	C3—N2	1.354 (7)
Mo3—O9	1.714 (3)	C3—H3	0.9300
Mo3—O1^i^	1.923 (3)	C4—C5	1.280 (9)
Mo3—O7	1.941 (3)	C4—N3	1.334 (7)
Mo3—O10	2.320 (3)	C4—H4	0.9300
Mo3—O12^i^	2.365 (3)	C5—N4	1.349 (10)
Cu1—O13	2.069 (3)	C5—H5	0.9300
Cu1—O13^i^	2.069 (3)	C6—N3	1.308 (8)
Cu1—O12	2.080 (3)	C6—N4	1.310 (10)
Cu1—O12^i^	2.080 (3)	C6—H6	0.9300
Cu1—O10^i^	2.096 (3)	N1—H1A	0.8600
Cu1—O10	2.096 (3)	N2—H2A	0.8600
As1—O14^i^	1.783 (3)	N3—H3A	0.8600
As1—O11	1.791 (3)	N4—H4A	0.8600
As1—O10	1.810 (3)		
			
O2—Mo1—O3	105.76 (15)	O11—As1—O10	98.94 (13)
O2—Mo1—O1	102.98 (14)	O11—As2—O12	100.25 (13)
O3—Mo1—O1	98.34 (13)	O11—As2—O15^i^	100.87 (15)
O2—Mo1—O4	94.47 (13)	O12—As2—O15^i^	98.60 (13)
O3—Mo1—O4	100.78 (13)	O14—As3—O15	99.93 (14)
O1—Mo1—O4	149.46 (12)	O14—As3—O13	99.56 (13)
O2—Mo1—O13	161.51 (13)	O15—As3—O13	99.65 (13)
O3—Mo1—O13	88.59 (12)	Mo1—O1—Mo3^i^	122.20 (14)
O1—Mo1—O13	85.98 (11)	Mo2—O4—Mo1	121.87 (14)
O4—Mo1—O13	70.99 (10)	Mo2—O7—Mo3	121.00 (14)
O2—Mo1—O12	91.53 (13)	As1—O10—Cu1	125.93 (14)
O3—Mo1—O12	162.04 (13)	As1—O10—Mo2	115.77 (13)
O1—Mo1—O12	72.51 (10)	Cu1—O10—Mo2	99.64 (10)
O4—Mo1—O12	82.27 (10)	As1—O10—Mo3	116.41 (13)
O13—Mo1—O12	75.58 (9)	Cu1—O10—Mo3	99.92 (11)
O6—Mo2—O5	105.65 (16)	Mo2—O10—Mo3	93.11 (9)
O6—Mo2—O7	103.19 (14)	As2—O11—As1	130.91 (17)
O5—Mo2—O7	96.23 (14)	As2—O12—Cu1	126.82 (14)
O6—Mo2—O4	96.59 (13)	As2—O12—Mo1	117.47 (13)
O5—Mo2—O4	100.89 (13)	Cu1—O12—Mo1	98.60 (10)
O7—Mo2—O4	149.22 (11)	As2—O12—Mo3^i^	116.78 (13)
O6—Mo2—O10	90.01 (13)	Cu1—O12—Mo3^i^	98.95 (10)
O5—Mo2—O10	162.31 (13)	Mo1—O12—Mo3^i^	91.05 (9)
O7—Mo2—O10	71.73 (10)	As3—O13—Cu1	126.18 (14)
O4—Mo2—O10	85.07 (11)	As3—O13—Mo1	115.12 (13)
O6—Mo2—O13	160.76 (13)	Cu1—O13—Mo1	99.30 (10)
O5—Mo2—O13	91.16 (13)	As3—O13—Mo2	117.13 (13)
O7—Mo2—O13	83.77 (11)	Cu1—O13—Mo2	99.54 (11)
O4—Mo2—O13	70.57 (10)	Mo1—O13—Mo2	93.50 (10)
O10—Mo2—O13	75.00 (9)	As3—O14—As1^i^	132.63 (17)
O8—Mo3—O9	105.69 (16)	As2^i^—O15—As3	130.07 (17)
O8—Mo3—O1^i^	97.38 (14)	N2—C1—N1	108.0 (5)
O9—Mo3—O1^i^	103.31 (14)	N2—C1—H1	126.0
O8—Mo3—O7	100.96 (14)	N1—C1—H1	126.0
O9—Mo3—O7	95.87 (13)	C3—C2—N1	107.2 (5)
O1^i^—Mo3—O7	148.71 (11)	C3—C2—H2	126.4
O8—Mo3—O10	92.78 (13)	N1—C2—H2	126.4
O9—Mo3—O10	159.45 (13)	C2—C3—N2	107.0 (5)
O1^i^—Mo3—O10	82.70 (11)	C2—C3—H3	126.5
O7—Mo3—O10	71.35 (10)	N2—C3—H3	126.5
O8—Mo3—O12^i^	163.96 (13)	C5—C4—N3	109.5 (6)
O9—Mo3—O12^i^	88.23 (13)	C5—C4—H4	125.2
O1^i^—Mo3—O12^i^	71.15 (10)	N3—C4—H4	125.2
O7—Mo3—O12^i^	85.17 (11)	C4—C5—N4	106.7 (7)
O10—Mo3—O12^i^	74.99 (9)	C4—C5—H5	126.7
O13—Cu1—O13^i^	180.0	N4—C5—H5	126.7
O13—Cu1—O12	86.42 (10)	N3—C6—N4	107.7 (6)
O13^i^—Cu1—O12	93.58 (10)	N3—C6—H6	126.2
O13—Cu1—O12^i^	93.58 (10)	N4—C6—H6	126.2
O13^i^—Cu1—O12^i^	86.42 (10)	C1—N1—C2	108.8 (4)
O12—Cu1—O12^i^	180.0	C1—N1—H1A	125.6
O13—Cu1—O10^i^	94.21 (10)	C2—N1—H1A	125.6
O13^i^—Cu1—O10^i^	85.79 (10)	C1—N2—C3	109.1 (5)
O12—Cu1—O10^i^	86.14 (10)	C1—N2—H2A	125.5
O12^i^—Cu1—O10^i^	93.86 (10)	C3—N2—H2A	125.5
O13—Cu1—O10	85.79 (10)	C6—N3—C4	107.6 (6)
O13^i^—Cu1—O10	94.21 (10)	C6—N3—H3A	126.2
O12—Cu1—O10	93.86 (10)	C4—N3—H3A	126.2
O12^i^—Cu1—O10	86.14 (10)	C6—N4—C5	108.5 (5)
O10^i^—Cu1—O10	180.0	C6—N4—H4A	125.8
O14^i^—As1—O11	99.33 (14)	C5—N4—H4A	125.8
O14^i^—As1—O10	99.24 (13)		

Symmetry codes: (i) −*x*+2, −*y*, −*z*.

### Hydrogen-bond geometry (Å, °)

**Table:**

*D*—H···*A*	*D*—H	H···*A*	*D*···*A*	*D*—H···*A*
N1—H1A···O4^ii^	0.86	1.81	2.664 (5)	173
N2—H2A···O3	0.86	1.99	2.748 (5)	146
N2—H2A···O9^iii^	0.86	2.42	3.020 (5)	127
N3—H3A···O7^iii^	0.86	2.09	2.867 (6)	150
N4—H4A···O2^iv^	0.86	2.00	2.834 (6)	165

Symmetry codes: (ii) *x*, −*y*+1/2, *z*+1/2; (iii) *x*−1, *y*, *z*; (iv) *x*, *y*, *z*+1.
